# Perinatal Micro-Bleeds and Neuroinflammation in E19 Rat Fetuses Exposed to Utero-Placental Ischemia

**DOI:** 10.3390/ijms20164051

**Published:** 2019-08-20

**Authors:** Ashtin B. Giambrone, Omar C. Logue, Qingmei Shao, Gene L. Bidwell, Junie P. Warrington

**Affiliations:** 1 Department of Neurology, University of Mississippi Medical Center, Jackson, MS 39216, USA; 2 Department of Cell and Molecular Biology, University of Mississippi Medical Center, Jackson, MS 39216, USA

**Keywords:** micro-bleeds, cerebral cytokines, preeclampsia, microglia

## Abstract

Offspring of preeclampsia patients have an increased risk of developing neurological deficits and cognitive impairment. While low placental perfusion, common in preeclampsia and growth restriction, has been linked to neurological deficits, a causative link is not fully established. The goal of this study was to test the hypothesis that placental ischemia induces neuroinflammation and micro-hemorrhages *in utero*. Timed-pregnant Sprague Dawley rats were weight-matched for sham surgery (abdominal incision only) or induced placental ischemia (surgical reduction of utero-placental perfusion (RUPP)); *n* = 5/group on gestational day 14. Fetal brains (*n* = 1–2/dam/endpoint) were collected at embryonic day (E19). Placental ischemia resulted in fewer live fetuses, increased fetal demise, increased hematocrit, and no difference in brain water content in exposed fetuses. Additionally, increased cerebral micro-bleeds (identified with H&E staining), pro-inflammatory cytokines: IL-1β, IL-6, and IL-18, eotaxin (CCL11), LIX (CXCL5), and MIP-2 (CXCL2) were observed in RUPP-exposed fetuses. Microglial density in the sub-ventricular zone decreased in RUPP-exposed fetuses, with no change in cortical thickness. Our findings support the hypothesis that exposure to placental ischemia contributes to microvascular dysfunction (increased micro-bleeds), fetal brain inflammation, and reduced microglial density in proliferative brain areas. Future studies will determine whether *in utero* abnormalities contribute to long-term behavioral deficits in preeclampsia offspring through impaired neurogenesis regulation.

## 1. Introduction

Preeclampsia (PE), a hypertensive disorder of pregnancy, is characterized by new onset hypertension with proteinuria, or in the absence of proteinuria, symptoms of other organ damage affecting kidney(s), the liver, or the brain [1]. Because PE is associated with increased risk of morbidity and mortality for the mother and offspring, PE contributes significantly to an increased public health burden. There is compelling evidence that exposure to PE has lasting effects on the offspring′s cognitive abilities. Offspring of PE patients go on to have lower IQ scores at 3 years of age [2], have impaired working memory [3], and have other neurobehavioral impairments [4,5] that progressively exacerbate cognitive impairment into the geriatric years [6,7]. Even though the underlying pathophysiological mechanisms are not known, placental insufficiency is believed to play a critical role in these poor neurodevelopmental outcomes [8].

In addition to being the source of the maternal syndrome [9], the dysfunctional placenta fails to meet the metabolic demands of the developing brain [10], resulting in clinical manifestations of neurodevelopmental disorders [11]. Specifically, offspring of PE-complicated pregnancies have a 32% increased risk of autism spectrum disorders [12,13,14]. Moreover, PE is an independent risk factor for long-term neuropsychiatric morbidity in the offspring [15] demonstrating that exposure to maternal factors and/or maternal vascular malperfusion [16] have lasting impacts on learning and memory-function in the offspring. The underlying pathophysiological mechanisms are not fully known.

One potential mechanism could be reduced blood flow to the developing placenta and fetus, a finding in some PE-complicated pregnancies. Indeed, studies report increased expression of hyoxia-inducible factor 1 alpha (HIF-1α) mRNA in placentas from women with preeclampsia [17,18]. The rodent placental ischemia model, induced by surgically reducing utero-placental perfusion pressure (RUPP), is well characterized and shares numerous characteristics with the PE patient. For example, RUPP rats have increased mean arterial blood pressure, with or without proteinuria [19], increased inflammatory cytokines [20,21], and increased anti-angiogenic factors [22,23]. Studies from members of our group have shown that RUPP dams have evidence of cerebrovascular abnormalities [24,25]; however, the impact of placental ischemia on the developing fetal cerebrovasculature has not been investigated. Additionally, whether placental ischemia induces neuroinflammation in the developing brain is not known.

In this study, we induced and modeled placental malperfusion by using the well-established rat model of placental ischemia [26], and determined the effect of five days of ischemia on fetal cerebral micro-bleeds and neuroinflammation. We measured the number of micro-hemorrhages as a marker of micro-bleeds and vascular function, brain water content as a measure of cerebral edema, and cytokine levels and microglia changes to assess neuroinflammation in embryonic day (E19) rat brains.

## 2. Results

### 2.1. General Characteristics and Pregnancy Outcomes: 

**Figure 1:**
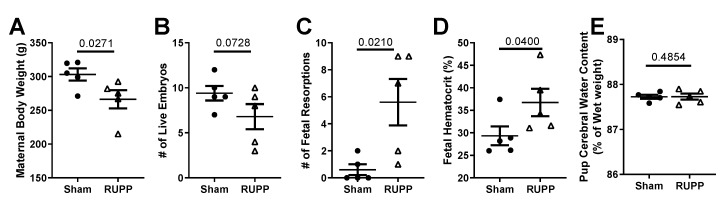
General characteristics of dams and fetuses subjected to placental ischemia. Dams had (**A**) reduced body weight, (**B**) reduced numbers of live fetuses, and (**C**) increased fetal demise at gestational day (GD) 19 compared to the sham controls. Fetuses subjected to placental ischemia had (**D**) increased hematocrits and (**E**) no change in brain water content. Values for individual rats (*n* = 5 dams per group) are shown along with the Mean ± SEM. Fetal hematocrit and pup brain water content represent the mean of 1–2 pups/dam (*n* = 5 dams). Differences between groups were analyzed using an unpaired *t*-test.

At gestational day (GD) 19, dams (*n* = 5 per group) subjected to placental ischemia had reduced body weight (266.5 ± 13.5 g) compared to sham-operated (control) pregnant rats (303.1 ± 9.1 g; *p* = 0.027; Figure 1A). 

A key characteristic of placental ischemia, induced using the RUPP procedure, is fetal demise in the form of increased fetal resorptions [19]. We therefore counted the number of live versus resorbed fetuses present at GD 19. Dams subjected to placental ischemia had a trend for fewer live fetuses (7 ± 1 in RUPP group versus 9 ± 1 in the sham control; *p* = 0.073; Figure 1B) and more fetal resorptions (6 ± 2 in RUPP group versus 1 ± 0 in the sham control; *p* = 0.021; Figure 1C) compared to the sham controls. This demonstrates that we successfully induced placental ischemia in the dams. We then assessed the effect of placental ischemia on the developing fetus. Because placental ischemia leads to reduced blood flow to the fetal-placental unit, we hypothesized that fetuses would have evidence of systemic hypoxia. We, therefore, measured pups’ hematocrits and found an increase in the hematocrits (36.7% ± 3.0% versus 29.3% ± 2.1% in sham) of fetuses exposed to placental ischemia (*p* = 0.040; Figure 1D). There was no difference in fetal brain water content between the groups (87.73% ± 0.04% in sham versus 87.73% ± 0.07% in RUPP-exposed; *p* = 0.485; Figure 1E), suggesting no cerebral edema. Additionally, in this cohort, we found no differences in maternal blood pressure between the groups (98 ± 4 mmHg in sham versus 99 ± 5 mmHg in RUPP; *p* = 0.405). Thus, our findings are due to utero-placental ischemia, independent of elevated blood pressure.

### 2.2. Micro-Hemorrhage in Fetal Brains 

**Figure 2:**
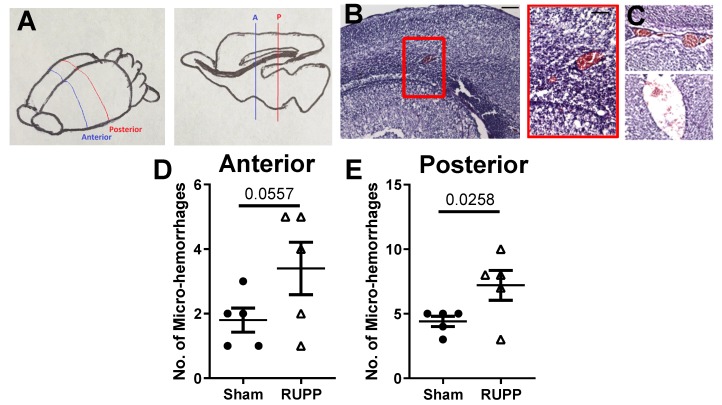
Placental ischemia exposure leads to increased number of micro-bleeds in brains of exposed fetuses. (**A**) Schematic of the regions where coronal sections were collected. (**B**) Representative images of fetal brains showing micro-bleeds in the cortex and (**C**) ventricles. Number of micro-bleeds in the (**D**) anterior (**E**) posterior slices of fetal brains. Points represent average micro-bleeds from 1–2 pups per dam (n = 5 dams per group). Mean ± SEM is also depicted. Data were analyzed using unpaired *t*-test and *p*-values are indicated.

Using H&E staining, red blood cells can be visualized by their red staining in tissues. Figure 2A shows the location of brain slices used to quantify micro-bleeds. Figure 2B,C show representative micro-bleeds observed in the parenchyma (2B, cortex) and lateral and third ventricles (2C).

We counted the number of micro-bleeds and found significantly higher numbers of micro-hemorrhages in the brains of fetuses exposed to placental ischemia in the posterior sections, and a trend for increased micro-bleeds in the anterior slices of the brain. In the anterior slices, RUPP-exposed fetuses had 3.4 ± 0.8 bleeds compared to the sham-exposed (1.8 ± 0.4 bleeds; *p* = 0.056; Figure 2D). In the posterior slices, sham-exposed fetuses had 4 ± 1 while RUPP-exposed fetuses had 7 ± 1 bleeds (*p* = 0.026, Figure 2E). Thus, exposure to placental ischemia almost doubled the incidence of fetal brain micro-bleeds *in utero*.

### 2.3. Inflammatory Profile in Fetal Brains 

**Figure 3:**
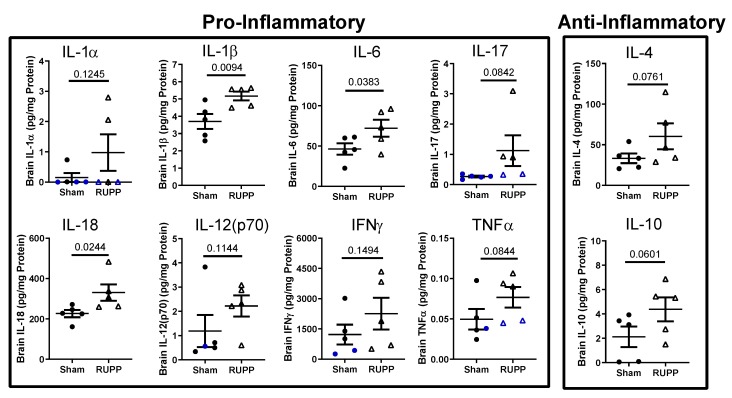
Placental ischemia leads to a shift towards a pro-inflammatory status in brains of exposed fetuses. A rat multiplex kit array of 27 cytokine/chemokine was used. Values were normalized to protein concentration. IL-1β, IL-6, and IL-18 increased significantly in fetal brains exposed to placental ischemia. Blue points represent extrapolated values (one value below the lowest detectable value and normalized to protein concentration). Not shown are: IL-2, IL-5, and IL-13. Values for individual rat fetuses (*n* = 5 fetuses per group) are shown along with the mean ± SEM. Only one fetal brain was collected per dam for cytokines/chemokines.

Due to placental ischemia inducing increased maternal, circulating and placental inflammatory cytokines [20,21,27], we hypothesized that the developing fetal brain may mirror the maternal pro-inflammatory environment. We therefore measured the levels of cytokines/chemokines in fetal brains exposed to normal pregnancy and placental ischemia. Out of 27 cytokines and chemokines, seven were undetectable or observed in only 1–2 fetal brains per group: EGF, G-CSF, GM-CSF, GRO/KC, IL-2, IL-5, and IL-13. We divided the remaining 20 cytokines/chemokines into pro-inflammatory/ cytotoxic, anti-inflammatory, and chemokines/growth factors [28]. The pro-inflammatory cytokines IL-1β, IL-6, and IL-18 increased significantly in fetal brains from placental ischemia-exposed pregnancies (Figure 3).

**Figure 4:**
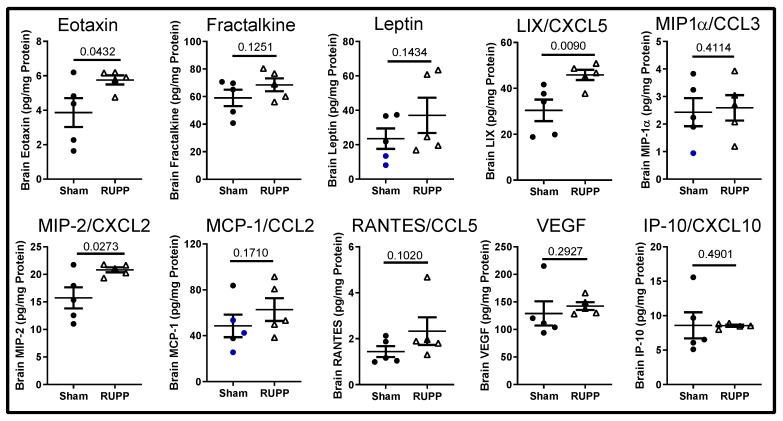
Changes in chemokines/growth factors in fetal brains. Placental ischemia exposure increased eotaxin, LIX, and MIP2 levels. Not shown are: EGF, G-CSF, GM-CSF, and GRO/KC. Blue points represent extrapolated values (one value below lowest detectable value and normalized to protein concentration). Values for individual fetuses (*n* = 5 fetuses per group) are shown along with the mean ± SEM. Data were analyzed using unpaired *t*-tests, and *p*-values are indicated.

There was a trend toward increased anti-inflammatory cytokines, IL-4 and IL-10. Lastly, the chemokines/growth factors eotaxin (CCL11), LIX/CXCL5, and MIP-2/CXCL2 increased significantly in fetal brains exposed to placental ischemia compared to sham-exposed (Figure 4).

These data demonstrate that placental ischemia exposure induces a pro-inflammatory environment in fetal brains *in utero*. Whether the cerebral inflammatory profile persists in the postnatal period is unknown. Thus, future studies will determine whether the fetal cerebral pro-inflammatory status is unique to the *in utero* environment or whether it persists postnatally.

**Figure 5:**
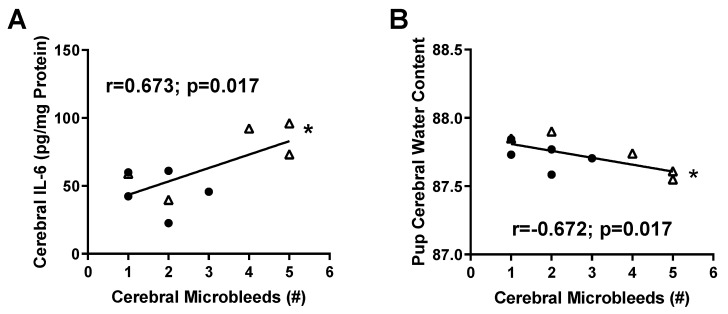
Correlation between number of micro-bleeds and IL-6 levels or brain water content. The number of micro-bleeds is (**A**) positively associated with fetal brain IL-6 levels and (**B**) negatively associated with fetal brain water content. Fetuses from same dam were used for the association analysis. Values for individual rats (*n* = 5 fetuses per group) are shown. Relationships between factors were analyzed using the Pearson correlation.

Because micro-hemorrhages are associated with a pro-inflammatory environment [29], we performed correlations to identify whether any factors were strongly associated with the number of micro-bleeds observed. Comparing fetuses from the same dam, we found that cerebral tissue IL-6 levels were positively associated with the number of micro-bleeds detected (Figure 5A; *r* = 0.673; *p* = 0.017). Surprisingly, although there were no differences in brain water content between the groups, fetal brain water content was negatively associated with the number of micro-bleeds (Figure 5B; r = −0.672; *p* = 0.017). 

### 2.4. Microglia Changes in Fetal Brains

**Figure 6:**
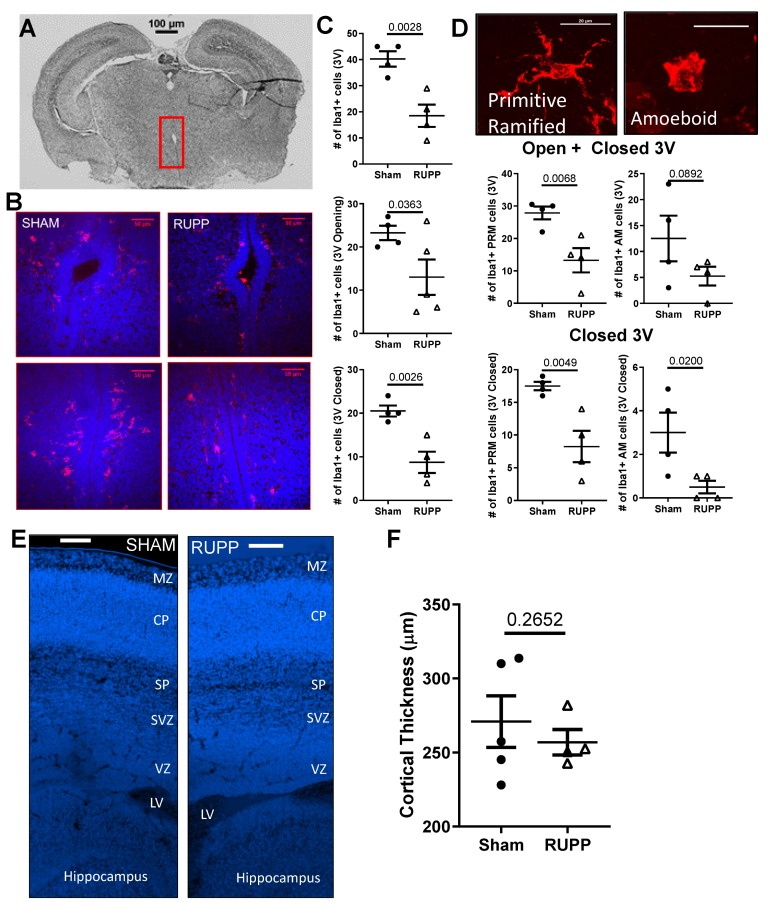
Changes in microglia at the proliferative areas (sub-ventricular zones—SVZ) of the 3rd Ventricle. (**A**) Representative image showing, at the brain-level, the section where the microglial analysis was conducted. Scale bar = 100 µm. (**B**) Representative images of Iba1 staining in the open region and closed region of the 3rd ventricle. Red—Iba1^+^ cells; Blue—DAPI^+^ nuclei. Scale bar represents 50 µm. (**C**) Decreased number of microglia in the SVZ of pups exposed to placental ischemia. (**D**) Examples of different microglia types observed (scale bar = 20 µm) and quantification of primitive ramified and amoeboid microglia in SVZ. (**E**) Representative images showing the different cortical layers in the embryonic day (E19) pup brain. Scale bar represents 100 µm. (**F**) Quantification of cortical plate thickness in fetuses exposed to sham or placental ischemia. Values from 1–2 pups were averaged per dam (*n* = 4–5 per group) and shown along with the mean ± SEM. Data were analyzed using unpaired *t*-tests, and *p*-values are indicated. MZ—marginal zone, CP—cortical plate, SP—subcortical plate, SVZ—sub-ventricular zone, VZ—ventricular zone, and LV—lateral ventricle.

Microglia are key producers of cytokines in the brain, so we assessed changes in microglial density and morphology in the brains of exposed fetuses. As reviewed in [30], microglia migrate from the ventricles and meninges during development, making the sub-ventricular zone (SVZ) the ideal region to quantify changes in microglial density. Representative images of a brain section and the third ventricle from sham and placental ischemia-exposed fetuses are shown in Figure 6A, B. We found fewer Iba1 + cells in the SVZ of the third ventricle (40 ± 3 in shams versus 19 ± 4; *p* = 0.003), and this reduction was observed both in the open (23 ± 2 in sham versus 13 ± 4; *p* = 0.036) and closed (21 ± 1 in shams versus 9 ± 2; *p* = 0.005) portion of the third ventricle (Figure 6C). We further characterized the microglia based on morphology into primitive ramified or amoeboid microglia [31] (Figure 6D) and counted these separately. We found a significant decrease in primitive ramified microglia in fetuses exposed to RUPP (13 ± 4 versus 28 ± 2 in Sham, *p* = 0.007; Figure 6D) and no change in the number of amoeboid microglia (13 ± 4 in Sham versus 5 ± 2 in RUPP, *p* = 0.089). We found no significant difference in cortical plate thickness (a potential indicator of neuronal density) between the fetuses exposed to normal or placental ischemic pregnancies (Figure 6E,F).

## 3. Discussion

Offspring born to preeclampsia patients have an increased risk of developing several neurological complications, including neurobehavioral abnormalities, cerebral palsy, cognitive impairment, and perinatal stroke [32,33,34]—the underlying mechanisms of which are not fully known. Here, we tested the hypothesis that utero-placental ischemia leads to *in utero* cerebrovascular changes, including micro-hemorrhages, which may contribute to future neurological complications. We found that indeed, placental ischemia in the pregnant female rat induces an increased number of cerebral micro-bleeds, a more pro-inflammatory cerebral-tissue environment, and decreased microglial density in the sub-ventricular zone of fetal brains *in utero*.

Like previous findings, the RUPP procedure led to decreased maternal body weight and increased fetal demise, demonstrating successful induction of placental ischemia [19,35]. Because we did not see an increase in blood pressure in this small cohort of rats, our findings are attributable to placental ischemia, independent of hypertension. We hypothesized that as a result of reduced blood flow to the fetus, surviving fetuses would have evidence of hypoxia. We found significant increases in the hematocrits of fetuses exposed to placental ischemia, suggesting systemic hypoxia. Whether the increased fetal hematocrits were associated with increased angiogenesis through vascular endothelial growth factor (VEGF) in our model is not known. While this possibility cannot be ruled out, our finding of no difference in VEGF levels in the brain homogenates of exposed fetuses suggests that cerebral angiogenesis may not be different between the groups. VEGF also increases vascular permeability and could increase vessel leakage, causing extravasation of plasma proteins. Thus, as a measure of vascular dysfunction, we quantified micro-bleeds within tissue slices.

The incidence of cerebral micro-bleeds was significantly higher in the placental ischemia-exposed group compared to the sham control group. Micro-bleeds are thought to occur when blood vessels are structurally damaged [36]. Thus, an increased incidence of micro-bleeds is consistent with ongoing vascular damage in the brains of exposed fetuses. Cerebral micro-bleeds can lead to long-term neurological damage, including cognitive and motor deficits [36]. Vascular damage and increased blood-brain barrier permeability can result in increases in brain water content; therefore, we hypothesized that fetuses exposed to placental ischemia would have increased cerebral water content, a crude marker of cerebral edema. Contrary to our hypothesis, we found no difference in brain water content between fetuses exposed to normal pregnancy and those exposed to placental ischemia. Even more interesting is the finding of a negative association between the number of cerebral micro-bleeds and brain water content. A recent study reported that micro-bleeds are observed in high-altitude-induced injury long after cerebral edema has resolved [31]. Thus, our finding of no difference in brain water content even in the presence of micro-bleeds could be an indication of a resolution of edema. We do not know whether different fetal or postnatal time-points would yield similar results and this is an area for future investigation. Additionally, we assessed water content in the entire fetal brain and could have missed regional changes in water content as a result.

Maternal inflammation and cerebral micro-bleeds are associated with increases in neuroinflammation. Therefore, we assessed the levels of different cytokines/chemokines in brain homogenates of exposed fetuses. We found increases in the pro-inflammatory cytokines, IL-1β, IL-6, and IL-18, and a trend for increased levels of TNFα and IL-17 in brains of fetuses exposed to placental ischemia. Additionally, IL-10 and IL-4 levels tended to increase in brains of fetuses exposed to placental ischemia. Those data suggest two possibilities. The first possibility is that there is an increased transfer of maternal inflammatory factors across the placental barrier into the fetal circulation. This is plausible since preeclampsia patients have increased circulating levels of tumor necrosis factor alpha (TNFα) [20], interleukin (IL)-2 [37,38], IL-6 [21], and IL-17 [39,40,41]. Placental ischemic rats also have increased levels of inflammatory cytokines in the circulation and cerebrospinal fluid [20,21,27,42,43]. We have not assessed changes in placental barrier permeability in the RUPP model of placental ischemia, and this is an area of ongoing investigation. The second possibility is that there is increased local production of these cytokines in the fetal brains following exposure to placental ischemia. 

A key finding in this study is that exposure to placental ischemia leads to increased cerebral levels of the pro-inflammatory cytokine, IL-1β in utero. There is evidence that placentas from preeclampsia patients secrete more IL-1β compared to normotensive placentas [44]. Additionally, infusion of IL-1β into the brains of young rats induces blood-brain barrier breakdown [45]. Taken together, increased tissue levels of IL-1β may play a deleterious role at the blood-brain barrier, contributing to structural damage and subsequent cerebral micro-bleeds. Whether increased IL-1β has a causal role in increased cerebral micro-bleeds in placental ischemia-exposed fetuses will be assessed in future studies.

Our multi-plex cytokine analysis also revealed increased levels of IL-6 and IL-18 in the brains of exposed fetuses. Not only was cerebral IL-6 increased in response to placental ischemia, but fetal brain IL-6 levels positively correlated to the number of cerebral micro-bleeds. Our findings are consistent with reports that serum levels of both IL-6 and IL-18 are higher in patients with cerebral micro-bleeds compared to those without [46]. Another study found that in patients with ischemic cerebrovascular disease, IL-6 was associated with an increased risk for cerebral micro-bleeds in an elderly, community-based cohort [47]. Furthermore, in the developing brain, micro-bleeds were observed in pups exposed to lipopolysaccharide, coupled with intrauterine ischemia, mainly if they were vaginally delivered [48], suggesting that *in utero* insults make blood vessels more susceptible to injury. Taken together, those studies and our current findings support the hypothesis that maternal inflammation, induced by placental ischemia, contributes to weakened cerebral vessels, making them more susceptible to blood-brain barrier (BBB) disruption and cerebral micro-bleeds.

Previously, we reported that eotaxin (CCL11) is increased in the cerebrospinal fluid of placental ischemic dams [42,49]. In the current study, we found increased levels of eotaxin in the brains of fetuses exposed to placental ischemia. The consequences and source of fetal brain eotaxin levels were not directly investigated in this study, although eotaxin has been shown to promote glutamate-induced neurotoxicity [50]. Importantly, CCL11 has been shown to directly regulate neurogenesis [51], such that increased circulating eotaxin was associated with reduced neurogenesis in aged mice and infusion of CCL11 into young mice resulted in decreased neurogenesis and cognitive impairment. Thus, increased eotaxin levels in the brains of fetuses exposed to placental ischemia suggest that neurogenesis may be impaired.

Microglia are essential during normal brain development and have important roles in pruning synapses, phagocytizing excess neuronal progenitor cells, and regulating the number of neurons at each stage of development [52]. Thus, changes in microglial numbers at late gestation could predict later neurological function. Microglia are also involved in secreting pro-inflammatory cytokines; thus, increased density of microglia could contribute to increases in local tissue inflammation. Following strokes, immune cells (microglia and macrophages) are activated by cytokines and chemokines, and migrate to damaged areas to remove dead neural cells. Those immune cells, induced by a chronic inflammatory environment, may become over-activated and produce large amounts of pro-inflammatory cytokines, disrupting neurogenesis and the BBB [53]. The presence of a pro-inflammatory environment and vascular damage in the brains of fetuses exposed to placental ischemia suggest that microglia may be activated. We therefore hypothesized that brains from exposed fetuses would have an increased density of microglia. Contrary to our hypothesis, we found that placental ischemia exposed fetuses had a decreased density of microglia in the SVZ of the third ventricle. Microglia in that region are vital for neurogenesis and oligodendrogenesis during fetal development and throughout life [52]. Reduced microglial density in the SVZ of placental ischemia-exposed fetuses may predict deficits in neurogenesis and oligodendrogenesis affecting the central nervous system long term. This possibility is a subject of ongoing investigation.

There is evidence that in both rats and mice, microglia protect neonatal brains from injury after ischemic stroke, and that depletion of microglia led to worse outcomes [54]. Thus, increased cerebral micro-hemorrhages, as occurred in exposed fetuses, coupled with fewer microglia, may indicate more severe damage/outcomes. During development, microglia can be disturbed by cytokines and chemokines released under conditions of maternal inflammation [55] seen in placental ischemia. 

To our knowledge, this is the first study reporting the impact of placental ischemia on cerebral micro-bleed incidence and neuroinflammation in the offspring in utero. The inclusion of male and female offspring in all analyses is a strength of the current study; however, we were unable to assess sex differences in our endpoints. Ongoing and future studies are now utilizing genotyping for sex to specifically assess sex differences. Additionally, this study utilized only one time-point (E19) and we are therefore unable to extend our findings to the postnatal period. Because we used brain homogenates to assess the inflammatory status, we were unable to address regional changes in expression of the different cytokines, chemokines, and growth factors assessed. We limited our microglial analysis to the SVZ associated with the third ventricle for this study; however, analysis of microglial changes in other brain regions will be important. 

In conclusion, the rat placental ischemia model induces increased cerebral micro-hemorrhages in the developing brain in utero and may be a good model to assess the etiology of cerebral micro-hemorrhages associated with preeclampsia-complicated pregnancies. Our data suggest that a hypoxic environment in the fetus, induced by reducing maternal utero-placental perfusion, induces abnormalities in cerebrovascular structure, increased blood-brain barrier disruption and micro-bleeds in the developing fetal brain. This is associated with a pro-inflammatory environment and reduced microglial density in proliferative brain regions, and may underlie the increased neurodevelopmental abnormalities observed in offspring born to preeclampsia patients. Additional studies are required to elucidate the underlying causes of these observations and to establish whether there are time-dependent differences in the outcome measures.

## 4. Materials and Methods 

### 4.1. Animals

Female timed-pregnant Sprague Dawley rats were obtained from Harlan Laboratories and arrived on gestational day (GD) 11. For timed pregnant rats from Harlan Laboratories, the day of vaginal plug detection was considered gestational day 0. Pregnant rats were housed singly after surgery (GD 14) and had continuous access to standard rodent chow and tap water. One to two (1–2) fetuses per dam per endpoint were randomly selected at E19 from 5 dams per group. Both male and female fetuses were included in all analyses; however, the sex of individual fetuses was not determined. Rats were maintained on a 12 h light and 12 h dark cycle. All animal procedures were approved by the University of Mississippi Medical Center’s Institutional Animal Care and Use Committee before animal procedures commenced (1379A, June 16 2016).

### 4.2. Placental Ischemia Induction

On GD 14, rats were anesthetized using isoflurane and an abdominal incision was made. The utero-placental unit was exteriorized and a silver clip (0.203 mm) was placed on the abdominal aorta (below the kidneys and above the bifurcation). Silver clips (0.1 mm) were also placed on both uterine artery branches between the ovaries and the first pup. This procedure induces placental ischemia by reducing utero-placental perfusion pressure (RUPP). Pregnant rats in the sham group were treated similarly, in that, an abdominal incision was made, the uterine horn was exteriorized, and vessels were manipulated without clip placement. Carprofen (5 mg/kg) was used as an analgesic in both groups.

### 4.3. Carotid Surgery and Blood Pressure Measurement

On GD18, rats were anesthetized using isoflurane anesthesia and the left carotid artery was isolated and cannulated using pre-made saline-filled catheters. Catheters were secured and exteriorized at the nape of the neck. The incision was closed and secured using Vetbond. The following morning, rats were placed in restrainer cages and catheters were connected to a pressure transducer. After 30 min of acclimation, blood pressure was recorded for 30 min using LabChart software. The mean arterial pressure was calculated for the duration of 30 min.

### 4.4. Harvest and Collection of Tissues

On GD 19, pregnant rats were anesthetized using isoflurane, and an abdominal incision was made. After exteriorization of the fetal-placental unit, maternal blood was collected from the abdominal aorta. Dams were euthanized by removal of the heart. The number of live and resorbed pups were counted. Fetuses were euthanized by decapitation. Trunk blood was collected from fetuses using micro-capillary tubes and hematocrit was noted. Fetal heads were processed differently depending on endpoints. For brain water content, brains were removed, weighed, and then dried for 48 h at 60 °C. Brain water content was calculated as a percentage ((wet weight − dry weight)/ wet weight). Brains for molecular analyses were removed and flash frozen in liquid nitrogen, followed by storage in a −80 °C freezer until processing. Brains for immunofluorescence staining or histology were kept within the skull and placed in 4% paraformaldehyde at 4 °C overnight. The following day, brains were removed from the skull and placed back in 4% paraformaldehyde overnight. Brains were then transferred to a 30% sucrose solution at 4 °C for 72 h and then embedded in Cryogel, and frozen at −80 °C until sectioning (1–2 fetal brains per mold). Brains were sectioned at 20 µm thickness, transferred directly to slides, and stored at −20 °C until staining.

### 4.5. Micro-Bleed Detection

Slides were washed and stained using hematoxylin and eosin following the manufacturer’s directions. Slides were then cover-slipped and imaged using light microscopy. Micro-bleeds were identified as red cells within the parenchyma and outside of the blood vessel lumen or within the ventricles. Micro-bleeds were counted by an investigator blinded to the groups (ABG) in sections from the anterior and posterior part of the brain (Figure 2A). The number of micro-bleeds from two slices per region was averaged per fetus and further averaged per dam. 

### 4.6. Fetal Brain Multiplex Array

To determine the cerebral inflammatory profile of exposed fetuses, a separate group of brains (*n* = 1 pup per dam) were homogenized in RIPA buffer, and protein concentration was measured using the BCA kit. Equal volumes (25 µL) of sample were loaded into 96 well-plates and incubated with magnetic beads, pre-mixed to detect 27 cytokine/chemokines (Rat multiplex kit, Millipore Sigma, Burlington, MA, USA). Samples were run alongside standards and kit controls in duplicate. The observed concentration was calculated using the standard curve generated and normalized to the protein concentration of the sample. Cytokine/chemokine concentration is, therefore, presented as pg/mg protein. 

### 4.7. Analysis of Microglia

Slides were washed and then blocked using normal donkey serum followed by rabbit anti-Iba1 polyclonal antibody (1:500; Wako; 019-19741) overnight at 4 °C. This Iba1 antibody recognizes the carboxy-terminal of the Iba1 protein and is specific to microglia/macrophages. Slides were washed and incubated for 2 h in donkey anti-rabbit TRITC (JacksonImmuno, West Grove, PA, USA; 131591) at room temperature. After washing, slides were mounted using Vectashield Mounting Media with DAPI and placed on coverslips. Images were captured using confocal microscopy with a 40X objective. Iba1^+^ cells were counted in the sub-ventricular zones (SVZ) associated with the third ventricle. A total of 2 sections per fetus were used for microglial assessment. Images of the third ventricle’s open and closed regions were captured from each fetal brain. Microglia totals from 1–2 pups were averaged per dam. Counting was done in a blind fashion. At E19, microglia have a different morphology from those in the adult brain; and amoeboid and primitive ramified microglia are most commonly observed [31]. We therefore counted the number of amoeboid and primitive ramified microglia in the SVZ. Image analysis was done using ImageJ (version 1.51j8; NIH). Cortical images were captured using a 10X objective, and the thickness of the cortical plate was analyzed using NIS Elements’ Analysis software (Nikon Instruments Inc., Melville, NY, USA; version 5.10.01). A total of 6 measurements per pup brain were obtained within the cortical plate and averaged per pup, and further averaged per dam.

### 4.8. Statistical Analysis

All statistical analyses were conducted using GraphPad Prism software (version 7). Differences between the sham-exposed and placental ischemia-exposed dams and fetuses were calculated using unpaired *t*-tests. For analyses where variances were different, we used Welch’s *t*-test with corrections for unequal variance. Pearson’s correlations were calculated to determine the association between various factors. Outlier tests (ROUT, Q = 1%) were conducted when visible outliers were suspected. For the microglia density data, two outliers (fetuses from the same dam from the sham-exposed group) were removed from analysis and are not shown. All graphs depict values for individual animals along with the mean ± SEM. The threshold for statistical difference was set at *p* < 0.05.

## 5. Conclusions

Placental ischemia, a common finding in preeclampsia-complicated pregnancies, leads to increased pro-inflammatory cytokines, increased micro-bleeds, and reduced microglia in proliferative zones of E19 fetal brains. Thus, our findings support the hypothesis that neuroinflammation, micro-vascular damage, and impaired microglia function may partly explain cognitive deficits that occur in offspring of preeclamptic pregnancies.

## Abbreviations

PE	Preeclampsia
RUPP	Reduced Uterine Perfusion Pressure
SVZ	Sub-ventricular zone
GD	Gestational day
BBB	Blood-brain barrier
